# Oncolytic adenovirus-mediated mda-7/IL-24 expression suppresses osteosarcoma growth and enhances sensitivity to doxorubicin

**DOI:** 10.3892/mmr.2021.12476

**Published:** 2021-10-04

**Authors:** Zongming Liu, Libo Xu, Hongping Yuan, Yang Zhang, Xiaona Zhang, Dongxu Zhao

Mol Med Rep 12: 6358-6364, 2015; DOI: 10.3892/mmr.2015.4180

Following the publication of this paper, it was drawn to the Editors’ attention by a concerned reader that the western blot assay data shown in Figs. 1B, 5A, 6A and C were strikingly similar to data appearing in different form in other articles by different authors. Owing to the fact that the contentious data in the above article were already under consideration for publication prior to its submission to *Molecular Medicine Reports*, the Editor has decided that this paper should be retracted from the Journal. After having been in contact with the authors, they agreed with the decision to retract the paper. The Editor apologizes to the readership for any inconvenience caused.

